# Metabolic potentials of the gut microbes in Antarctic krill (*Euphausia superba*)

**DOI:** 10.1128/msystems.00377-25

**Published:** 2025-08-07

**Authors:** ZhanFei Wei, Lihui Meng, Sheng Du, Fang Wang, Aijun Jiang, Rui Lu, Kaiqiang Liu, Xinliang Wang, Qingchang Xu, Xiuxia Mu, Liang Meng, ChangWei Shao

**Affiliations:** 1 State Key Laboratory of Mariculture Biobreeding and Sustainable Goods, Yellow Sea Fisheries Research Institute, Chinese Academy of Fishery Sciences, Qingdao, China; 2 School of Marine Science and Engineering, Qingdao Agricultural University 98431, Qingdao, China; 3 Key Laboratory of Sustainable Development of Polar Fishery, Ministry of Agriculture and Rural Affairs, Yellow Sea Fisheries Research Institute, Chinese Academy of Fishery Sciences, Qingdao, China; 4 International Center for Deep Life Investigation (IC-DLI), Shanghai Jiao Tong University 12474, Shanghai, China; 5 BGI Research, Sanya, China; Universidad Miguel Hernandez de Elche, San Juan de Alicante, Alicante, Spain

**Keywords:** *Euphausia superba*, gut microbial diversity, metabolic function, direct metagenomic, culture-enriched metagenomics

## Abstract

Antarctic krill (*Euphausia superba*) plays crucial roles in the Southern Ocean biological system, yet the ecological functions of their gut microbiota remain poorly understood. The study investigated the composition and diversity of the gut microbiota in *E. superba* using 16S rRNA gene sequencing, revealing a highly diverse and tissue-specific microbiota. Further investigation through direct metagenomics and culture-enriched metagenomics identified 12 metagenome-assembled genomes (MAGs), which might represent novel species based on their phylogenetic positions, average nucleotide identity (ANI), average amino acid identity (AAI), and relative evolutionary divergence (RED) values. These MAGs were grouped into two distinct clusters according to their completeness of metabolic modules. Although MAGs in cluster 2 showed lower read relative abundance compared to those in cluster 1, they exhibited a greater number of amino acid synthesis modules. The synthesis of glutathione, heme, ubiquinone, vitamins, and betaine by MAGs suggested potential roles in enhancing survival of *E. superba* in extreme environment. Moreover, the functional genes in the 12 MAGs had abundant transcripts indicating they exhibited high metabolic activity in the *E. superba* gut microbiota. These findings shed new light on the metabolic contributions of gut microbiota in *E. superba*.

**IMPORTANCE:**

This study provided a comprehensive characterization of the gut microbiome of *Euphausia superba*, revealing its diverse and functionally distinct microbial community. Furthermore, 12 metagenome-assembled genomes (MAGs) representing novel species were successfully identified. The metabolic analysis demonstrated that these microbes contribute to host nutrient acquisition by synthesizing essential amino acids and vitamins. The identification of antioxidant and osmoregulatory compound synthesis modules suggested that gut microbiota might facilitate the survival of *E. superba* in the harsh Antarctic environment. Those findings elucidated the host–microbe interactions in polar marine ecosystems and provided new insights into microbial contributions to host nutrient cycling and environmental adaptation in the Southern Ocean.

## INTRODUCTION

The Antarctic krill (*Euphausia superba*), an endemic species of Antarctica with 300–500 million tons, is recognized as the largest biomass of wild animals on Earth (1). The *E. superba* plays a crucial role in the Southern Ocean ecosystem, serving as a primary food source for a variety of marine predators, including fish, seals, and whales (2). As a primary producer-consumer link in the Southern Ocean food web, *E. superba* facilitates energy transfer and nutrient cycling between lower and higher trophic levels, thereby contributing to the overall stability of the ecosystem (3–5). Their production of large, carbon-rich, and rapidly sinking fecal pellets are crucial for the sequestration of carbon in the deep ocean. In highly productive regions such as the marginal ice zone, these pellets account for 17%–61% of the total carbon flux (6). Recent studies have highlighted the complex interactions between *E. superba* populations and their environment, particularly in the context of climate change (7, 8).

*E. superba* harbors a diverse and complex microbial community, forming a stable symbiotic network that constitutes a unique microecosystem. Studies have shown that the bacterial communities in different tissues of *E. superba* exhibit significant specificity, reflecting dynamic interactions with host (9). Furthermore, the composition and diversity of microbial communities are distinct from the surrounding environment, indicating that *E. superba* harbors specialized microbiota (9). These microbial communities also exhibit spatial structuring influenced by geographical distance, suggesting that environmental factors might shape microbial composition (10). Recent research has revealed that the gut microbiota in *E. superba* likely play significant roles in nutrient acquisition by enriching the host hydrolytic enzyme repertoire (11). Moreover, microbial isolates from *E. superba* are identified, revealing their ability to produce antimicrobial and cytotoxic compounds (12). Additionally, the *Brucella* sp. strain (WY7) and *Pseudoalteromonas* sp. WY3, both isolated from *E. superba*, could contribute to the host survival through secondary metabolite production (13, 14). However, the metabolic characteristics of *E. superba* gut microbiota remain largely unexplored, particularly in understanding how they assist the host in adapting to extreme environment.

During the 2022 Antarctic expedition aboard the Xue Long 2, a large number of *E. superba* were successfully collected in the Southern Ocean, aiming to investigate their gut microbiome. The study revealed the microbial diversity and identified the dominant phyla in the *E. superba* gut microbiota. In total, 12 metagenome-assembled genomes (MAGs) classified into these dominant phyla were identified, which might represent novel species based on their unique evolutionary indices. Furthermore, these MAGs were grouped into two distinct hierarchical clusters based on the completeness of metabolic modules. Microbes appeared to contribute to the host metabolism, potentially supplying amino acids and vitamins for *E. superba*. Additionally, microbes in both clusters were involved in the synthesis of glutathione, heme, and ubiquinone functioned as antioxidants that could help *E. superba* mitigate oxidative stress in harsh environmental conditions. Further metatranscriptomic analysis confirmed the active presence of these 12 dominant bacterial species in the *E. superba* gut microbiota. By exploring the metabolic characteristics of these microbes, the findings improved understanding of specific diversity of the gut microbiota and shed light on the metabolic contributions of these microbes to *E. superba*.

## MATERIALS AND METHODS

### Sample collection and sequencing

**Fig 1:**
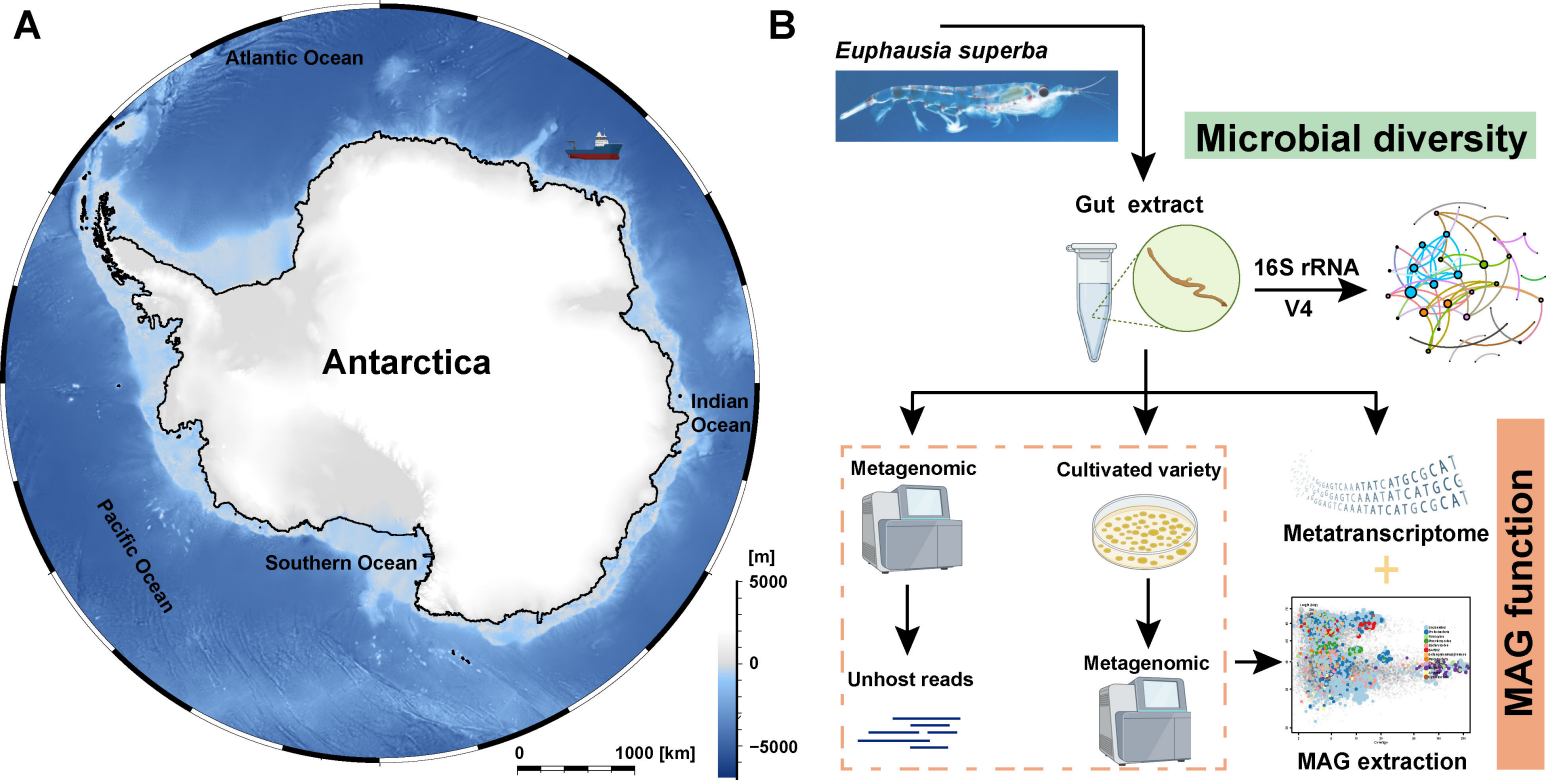
Sample location and schematic illustration of the study design. (**A**) The Antarctic krill (*Euphausia superba*) collected from the Southern Ocean (46.25°E, 66.60°S) by the Xue Long 2 in February 2022. (**B**) *E. superba* intestines extracted in the laboratory and subsequently used for 16S rRNA gene amplicon sequencing to analyze microbial composition and networks, as well as for metagenomic and cultivation-based genomic analyses to investigate ecological functions of metagenome-assembled genomes (MAGs).

*E. superba* specimens were collected from the Southern Ocean (46.25°E, 66.60°S) by the Xue Long 2 in February 2022 (Fig. 1A). The samples were rinsed with double-distilled water (ddH_2_O) and immediately preserved at −80°C onboard. Fifteen *E. superba* were further processed in the laboratory by washing with ddH_2_O, dissecting under a microscope, and rapidly excising the intestines. Among these, eight intestine samples were finely chopped and ground into a powder in a sterile mortar using liquid nitrogen for subsequent metagenomic analyses. Four intestine samples were pooled, homogenized, and evenly distributed into three seawater bacterial culture media (Zobell Marine Agar, Marine Agar 2216E, and Marine SC Medium) for a two-week incubation at 4°C. After incubation, colonies were harvested from each plate for culture-enriched metagenomic analyses (Fig. 1B).

Total DNA was extracted from these samples using the CTAB method and purified with the Qiagen Genomic-tip 100/G kit (Qiagen, USA). The DNA quality was assessed by gel electrophoresis, and the quantity was measured using a Qubit 2.0 Fluorometer (Life Technologies, USA). High-quality DNA (1 µg per sample), from eight intestines and three culture-enrichment samples, was prepared for whole-genome sequencing (WGS) libraries using the MGIEasy Universal DNA Library Prep Set (MGI, China) and then sequenced on the MGI2000 platform with PE × 150 bp to generate ~100 Gb per library.

The V4 regions of the 16S rRNA genes from eight intestine samples were amplified with universal primers 515F (5′- GTGCCAGCMGCCGCGGTAA-3′) and 806R (5′-GGACTACHVGGGTWTCTAAT-3′) to characterize the microbial composition. Libraries for 16S rRNA gene amplicons were prepared using the TruSeq Nano DNA LT Kit (Illumina, USA) and sequenced on the Illumina MiSeq platform with PE × 250 bp. The total RNA was isolated from three other *E. superba* intestines and used to construct cDNA libraries with the MGIEasy RNA Library Prep Set (MGI, China). Three cDNA libraries were sequenced on the Illumina platform with PE × 150 bp to generate ~50 Gb, respectively.

### Microbial composition and network analyses

The 16S rRNA genes amplicon was processed using QIIME2 pipeline (15). The amplicon sequence variants (ASVs) were classified using SILVA_138 database (16), excluding those associated with chloroplasts, mitochondria, and eukaryotes. The ASVs that appeared in more than 20% of the samples and had more than four counts in those samples were retained using Python (v3.8) script. The retained ASVs were utilized to assess microbial composition and diversity indices (ACE, Fisher, Shannon, and Simpson) in the *E. superba* gut microbiota. The 16S rRNA genes amplicon data from other tissues and the surrounding environments, previously reported in earlier studies, were re-analyzed using QIIME2 following the same processing pipeline (9). Then, the number of ASVs was normalized for further comparative analyses. The matrix of co-occurrence correlations for the ASVs was computed using psych package in R. These matrix values were imported into Gephi (v0.10.1) to construct a microbial community network, leveraging the built-in diversity analysis features (17).

### Metagenome assembly genomes and abundance profiling

The WGS reads from eight intestine samples underwent filtration to exclude the *E. superba* genome sequences using the BWA (v0.7.17) with default parameters (18). The host-deleted WGS, along with reads from three culture plates, were filtered using Fastp (v0.22.0) with parameters ‘-f 4 -F 4 -w 24 -c -q 20 -u 20-3 -l 50’ (19). The filtered reads were then assembled using MEGAHIT (v1.2.9) with the settings ‘-min-contig-len 1000 --presets meta-sensitive’ (20). The MetaWRAP (v1.2.1) facilitated the selection of MAGs with a completeness threshold above 50% and contamination below 10% (‘-c 50 -x 10’) (21). The CheckM (v1.2.2) was employed to assess the completeness and contamination of these MAGs (22). The taxa of MAGs were classified using the GTDB-Tk (v1.0.2) with default settings (23). The MAGs were deduplicated using dRep (v3.2.2) with a 95% identity threshold (24). Those direct metagenomic reads were mapped to MAGs using the BWA (v0.7.17) with default parameters (18). Then, the read relative abundance of each MAG in eight WGS data was calculated using CoverM (v0.6.1) with the ‘-m relative_abundance’ option (https://github.com/wwood/CoverM).

### Functional annotations and transcripts

The open reading frames (ORFs) from 12 MAGs were predicted using Prokka (v1.12) (25). Functional annotation of ORFs was performed by searching Kyoto Encyclopedia of Genes and Genomes (KEGG) and evolutionary genealogy of genes: Non-supervised Orthologous Groups (eggNOG) database with *e*-value <1*e*−5. The hydrolytic enzymes were identified through the carbohydrate-active enzymes (CAZy) database, employing the HMM, dbCAN_sub, and DIAMOND methods with default settings (26). Meanwhile, the KO number of *E. superba* genome was from our previous study (7). The completeness of material synthesis modules was assessed using the module-based results from KEGG MAPER (https://www.kegg.jp/kegg/mapper/). The hierarchical clusters and principal component analysis (PCA) were performed based on the completeness of metabolic modules using Euclidean method in vegan package of R. The total mRNA sequencing data were also filtered using the Fastp (v0.22.0) under ‘-w 24 -l 50’ (19). The expression level of functional genes from 12 MAGs was estimated by transcripts per million (TPM) values using CoverM (v0.6.1) with the ‘-m TPM’ option (https://github.com/wwood/CoverM).

### Phylogenetic distance analysis

A total of 106 Enterobacterales, 72 Pseudomonadales, 19 Rhodobacterales, 17 Actinomycetales, and 4 Pirellulales from the Genome Taxonomy Database (GTDB) were undertaken for phylogenomic analysis (Table S1). The 43 conserved aligned protein sequences in these genomes were screened by CheckM (v1.2.2) (22), and trimmed using trimAl (v1.4) with ‘-automated1’ parameters, respectively (27). The trimmed sequences of 43 conserved aligned proteins were concatenated and utilized to construct the phylogenomic tree using the IQ-TREE (v1.6.12) with maximum likelihood method (‘-m MFP -bb 1000 -alrt 1000’) (28). The phylogenomic trees facilitated the estimation of average nucleotide identity (ANI) and amino acid identity (AAI) between 12 gut and related MAGs using FastANI (v1.34) with ‘-minFraction 0.5’ (29) and CompareM (v0.1.2) (‘-e 1e-5 --sensitive’) (https://github.com/dparks1134/CompareM), respectively.

## RESULTS AND DISCUSSION

### Microbial composition and diversity

**Fig 2:**
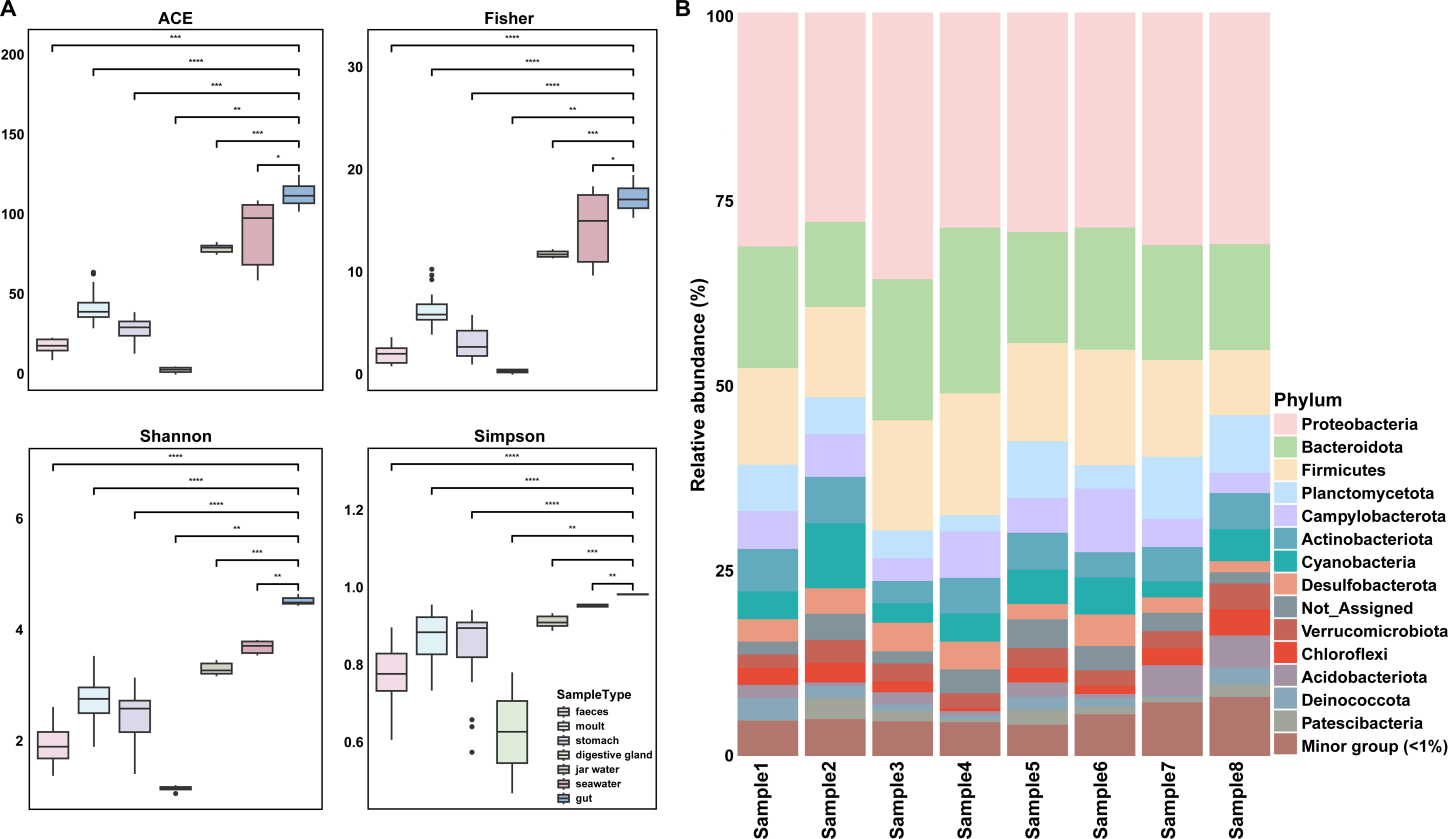
Microbial diversity and composition in the *E. superba* gut. (**A**)The alpha-index comprising ACE, Fisher, Shannon, and Simpson. * *P* < 0.05, **P < 0.01, ***P < 0.001, ****P < 0.0001. (**B**)The gut microbial composition at phylum observed in eight *E. superba*.

A total of 2,651 ASVs were initially collected during the analysis. After applying rigorous data filtration processes, 243 ASVs were retained for subsequent microbial community and diversity analyses. Rarefaction curves showed that the number of ASVs reached plateaus in all the samples, indicating that nearly all microbial species present in the *E. superba* gut microbiota were captured (Fig. S1). In comparison to previous study, the gut microbiota in *E. superba* harbored a significantly greater number of species than those identified in other sources, such as the stomach, feces, molt, digestive gland, and environmental water (Wilcox-test, *P* < 0.05), underscoring the tissue-specific variation in microbial composition (Fig. S2) (9). Diversity indices (ACE, Fisher, Shannon, and Simpson) further revealed the richness and evenness of the *E. superba* gut microbiota, highlighting the diverse gut microbial ecosystem (Fig. 2A). These tissue-specific microbial profile and elevated diversity suggested that the *E. superba* possessed unique gut microbiota, potentially shaped by the physiological requirements of the host.

The gut microbial communities of *E. superba* were predominantly composed of Proteobacteria, Bacteroidota, Firmicutes, Planctomycetota, Campylobacterota, Actinobacteriota, Cyanobacteria, Desulfobacterota, Verrucomicrobiota, Chloroflexi, Acidobacteriota, Deinococcota, and Patescibacteria. These phyla except for Patescibacteria were also identified as the core microbiome in the *E. superba* gut microbiota (Fig. 2B; Fig. S3). Proteobacteria accounted for 30.7% of the *E. superba* gut microbiota, consistent with their widespread dominance in marine ecosystems. This predominance highlighted the significance of Proteobacteria in the *E. superba* gut, likely attributed to their metabolic versatility and adaptation to the nutrient-rich environments (30). Other bacterial phyla, including Bacteroidota, Firmicutes, and Actinobacteriota, also showed abundance in other tissues, highlighting their prominence in the *E. superba* microbiomes (9, 10). High abundances of Proteobacteria, Bacteroidetes, Firmicutes, and Actinobacteriota were observed in the gut microbiome of marine mussels, playing key roles in energy storage during dietary stress and food scarcity (31). The Proteobacteria and Bacteroidota were also found to predominate in the Antarctic sponges microbiomes, regardless of their specific habitat (32). The predominance of Firmicutes had also been reported in bottlenose dolphins and fermenting sirenians, likely due to their ability to harvest energy and nutrients from ingested food (33). Therefore, these abundant microbial phyla could potentially support *E. superba* to thrive in unique extreme environment by contributing to essential metabolic pathways.

At the order level, the dominant taxa (>1%) included Pseudomonadales, Bacteroidales, Rhizobiales, Flavobacteriales, Campylobacterales, Chloroplast, Pirellulales, Chitinophagales, Holosporales, Burkholderiales, Peptostreptococcales_Tissierellales, Bacillales, Enterobacterales, Lachnospirales, Rhodobacterales, Oscillospirales, Sphingomonadales, Rickettsiales, Desulfobulbales, Verrucomicrobiales, Vicinamibacterales, Entomoplasmatales, Thermales, Planctomycetales, Lactobacillales, and Micrococcales (Fig. S4A). These orders, known for their roles in nutrient cycling, organic matter degradation, and secondary metabolite production, suggested that those microbes might support a range of ecological functions for host. Furthermore, a co-occurrence network analysis was performed to explore the non-random aggregation patterns of gut microbial communities in the *E. superba* (Fig. S4B; Table S2). Analysis of top 200 high-abundance ASVs revealed significant correlations among 181 microbial nodes, forming a network with 374 edges (Table S3). The strong interactions between microbial species (*ρ* > 0.8, *P* < 0.05) supported the presence of complex and interdependent relationships in the *E. superba* gut microbiota. The ASVs in the network primarily fall into 12 dominant bacterial phyla, with Proteobacteria accounting for 33.1% of all nodes. Those findings suggested strong interaction between Proteobacteria and other dominant phyla, underscoring their role in shaping the gut microbial community structure within *E. superba*.

### Sequencing and assembly of microbiomes

A total of 575 Gb of WGS data were collected from eight intestine samples, which included both host-derived and microbial reads. Following quality control and filtration processes to remove host-derived and low-quality reads, 17 Gb of high-quality reads were retained for co-assembly, resulting in the generation of 0.75 Gb of contigs for MAG extraction. The substantial size of the *E. superba* genome presented considerable challenges for metagenomic sequencing and data processing, as host-derived reads frequently constituted the majority of raw sequencing data (7). The obstacle was particularly pronounced in non-model organisms with large and complex genomes such as human, where accurately separating microbial reads from holobiont was essential for reliable metagenomic analysis (34). Therefore, the comprehensive filtering process, including removal of host-derived sequences and enrichment of microbial reads, was crucial for improving the accuracy of microbial genome assembly, functional annotation, and ecological characterization.

To in-depth investigate the *E. superba* gut microbiota, culture media were used to enrich microbes. 119 Gb (Zobell Marine Agar), 131 Gb (Marine Agar 2216E), and 138 Gb (Marine SC) WGS data were generated from three culture plates. Following quality control and filtration to remove low-quality sequences. The filtrated data were also assembled to result in the generation of 99 Mb, 85 Mb, and 75 Mb contigs for MAG extraction, respectively. By integrating culture enrichment with sequencing, culture-enriched metagenomics offered a powerful approach for studying microbiota (11, 12). The culture-enriched metagenomics had enhanced microbial genome assembly efficiency and facilitated the identification of new microbial species in human cystic fibrosis sputum (35). Similarly, culture-enriched metagenomics were performed on hadal sediment samples in the Mariana Trench to promote genomic understanding of hadal microbes (36). While culturing enrichment might capture microbes adaptable to specific media, the combination of multiple culture media, high-throughput sequencing, and rigorous data processing greatly facilitated a more comprehensive profiling of the gut microbiota in *E. superba*.

### Microbial genome reconstruction

Despite generating extensive WGS data from eight intestine samples, ~3% of the total reads mapped to microbial sequences. Only three MAGs were extracted from direct metagenomics just representing a small fraction of the gut microbiota. Additionally, 13 MAGs representing diverse phyla were recovered from the culture-enriched metagenomics, which might selectively enrich specific microbial populations. However, the combination of direct metagenomics and culture-enriched metagenomics enhanced the ability to capture a broader and more representative microbes, facilitating more comprehensive insight into the microbial ecology within the host (35). The 16 MAGs exhibited a completeness over 50% and contamination below 10%. After deduplication with 95% identity, 12 MAGs were retained for further analysis (Table 1). According to the Minimum Information about a Metagenome-Assembled Genome (MIMAG) standards, three of these MAGs met the criteria for high-quality with completeness exceeding 90% and contamination below 5%. The remaining MAGs were classified as medium-quality based on their completeness and contamination (37). The genome sizes of the 12 MAGs ranged from 1.3 Mb to 4.0 Mb, and their GC content varied between 37.5% and 64.4%. These metrics reflected the variability in genome sizes and genetic composition across the different gut microbes in the *E. superba*.

**TABLE 1:** Information of microbiota metagenome-assembled genomes (MAGs) in *E. superba* gut

ID	RED value	Completeness (%)	Contamination (%)	No. of genes	Genome size (bp)	GC content (%)
*Yoonia euphausia 2*	0.97	99.3	1.3	3,545	3,627,542	54.6
*Yoonia euphausia 1*	0.97	55.2	0	2,056	2,062,185	56.1
*Psychromonas euphausia*	0.98	51.7	0	1,132	1,340,474	37.5
*Psychrobacter euphausia 3*	0.99	61.1	2.9	1,491	1,815,146	42.7
*Psychrobacter euphausia 2*	0.99	65.9	8.9	2,028	2,566,887	42.3
*Psychrobacter euphausia 1*	0.99	55.5	3.5	1,600	1,861,438	43.0
*Pseudoalteromonas euphausia*	0.99	98.9	0.4	3,265	3,617,529	39.8
*Mariniblastus euphausia*	0.98	73.1	1.2	3,279	4,013,170	48.5
*Halioglobus euphausia 2*	0.98	92.0	5.7	3,009	3,320,685	52.2
*Halioglobus euphausia 1*	0.99	61.7	0.7	1,765	1,853,113	53.7
*Arthrobacter euphausia 2*	0.98	93.7	2.3	2,610	2,716,408	64.4
*Arthrobacter euphausia 1*	0.99	56.7	0	3,108	3,311,308	59.8

### Homology relationship analysis

**Fig 3:**
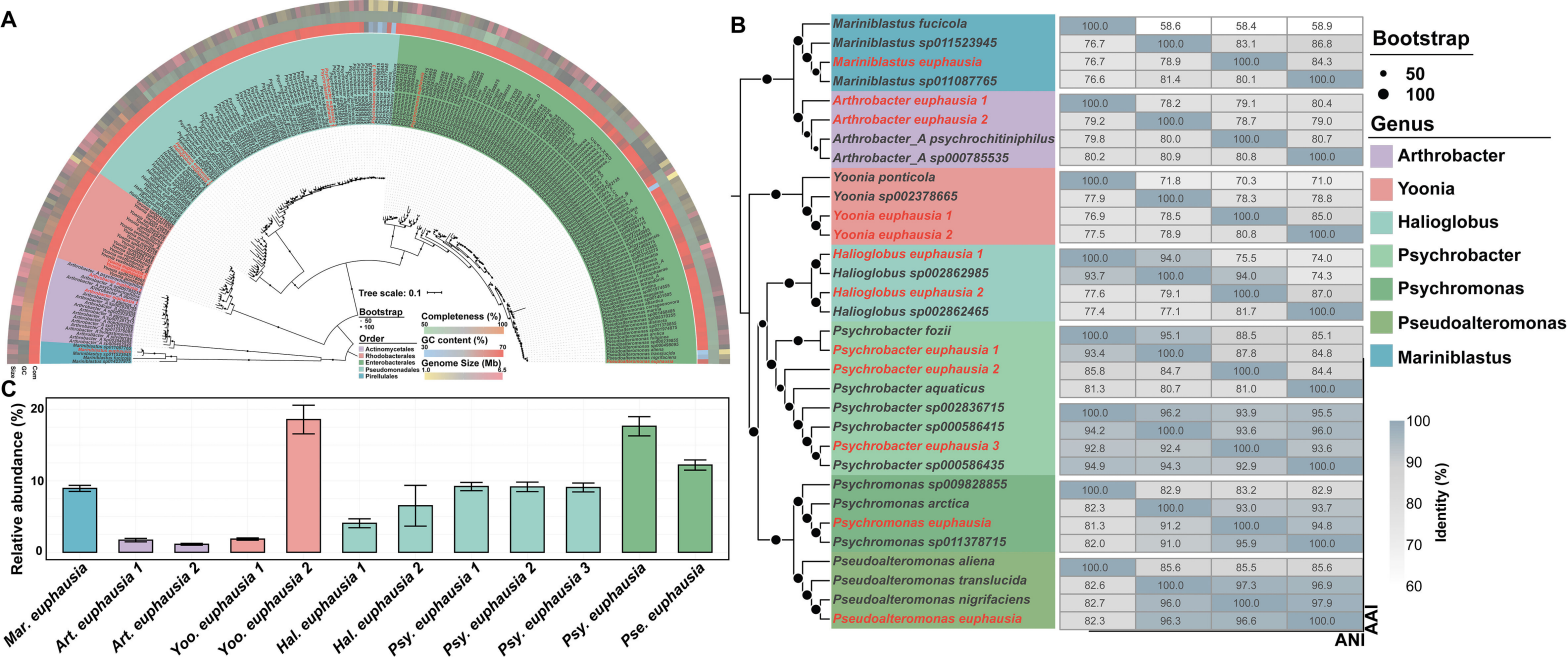
Phylogenomic tree, average nucleotide identity (ANI), average amino acid identity (AAI), and read abundance profiling of 12 MAGs. (**A**) The phylogenomic tree constructed using 43 conserved proteins, with the reference MAGs listed in Table S1. (**B**) The AAI and ANI values of the 12 MAGs. The species were selected based on genetic distance shown in panel A. (**C**) The read relative abundance of the 12 MAGs across the 8 WGS data.

Based on the taxonomic classification, 12 MAGs represented three major phyla commonly found in *E. superba* gut microbiota, including phyla Proteobacteria (9 MAGs), Actinobacteriota (2 MAGs), and Planctomycetota (1 MAG) (Fig. 3; Table 1). To determine the phylogenetic relationships of these microbes, a phylogenomic tree was constructed, revealing that 12 MAGs clustered into 5 distinct orders (Fig. 3A). Among them, *Mariniblastus euphausia* was closely related to *Mariniblastus sp011*087765 within the order Pirellulales. Although *Arthrobacter euphausia 1* and *Arthrobacter euphausia 2* both fall into the order Actinomycetales, they were positioned distantly in the tree. *Arthrobacter euphausia 1* occupied a single branch, while *Arthrobacter euphausia 2* was closely related to *Arthrobacter_A psychrochitiniphilus. Yoonia euphausia 1* and *Yoonia euphausia 2*, in the order Rhodobacterales, were clustered together with different branch lengths, forming distinct evolutionary branch from *Yoonia sp002378665*. In the order Pseudomonadales, *Halioglobus euphausia 1* was clustered with *Halioglobus sp002862985*, while *Halioglobus euphausia 2* was closely related to *Halioglobus sp002862465*, with each located on separate branches. Furthermore, *Psychrobacter euphausia 1* was clustered with *Psychrobacter fozii*, and *Psychrobacter euphausia 2* was closed to *Psychrobacter aquaticus*. While *Psychrobacter euphausia 3* showed a closer phylogenetic relationship with *Psychrobacter sp000586435*. Additionally, *Psychromonas euphausia* and *Pseudoalteromonas euphausia*, both in the order Enterobacterales, displayed distinct phylogenetic positions. *Psychromonas euphausia* was closely related to *Psychromonas sp011378715*, while *Pseudoalteromonas euphausia* was closely aligned with *Pseudoalteromonas nigrifaciens*.

The ANI and AAI values of the 12 MAGs were evaluated based on their phylogenetic positions (Fig. 3B). *Mariniblastus euphausia* showed ANI values below 80.1% and AAI values below 84.3%. The highest ANI values for *Arthrobacter euphausia 1* and *Arthrobacter euphausia 2* were 80.2% and 80.9%, with highest AAI values of 80.4% and 79.0%. *Yoonia euphausia 1* and *Yoonia euphausia 2* exhibited the highest ANI and AAI values of 80.8% and 85.0%. *Halioglobus euphausia 1* achieved ANI and AAI values of 93.7% and 94.0%. *Halioglobus euphausia 2* showed highest ANI and AAI values of 81.7% and 94.0%. Furthermore, *Psychrobacter euphausia 1* exhibited highest ANI and AAI values of 93.4% and 95.1%, while *Psychrobacter euphausia 2* showed highest ANI and AAI values of 85.8% and 88.5%. Additionally, the ANI and AAI values of *Psychrobacter euphausia 3* reached 92.9% and 93.9%. Finally, *Psychromonas euphausia* recorded highest ANI and AAI values of 95.9% and 94.8%, while *Pseudoalteromonas euphausia* showed highest ANI and AAI values of 96.6% and 97. 9%.

The phylogenetic tree distinctly positioned these MAGs within unique evolutionary branches, indicating significant divergence from known species. The phylogenetic inference was further supported by ANI and AAI values, which demonstrated low similarity to their related species. In addition, the relative evolutionary divergence (RED) values measured the divergence between the MAGs and known relatives (Table 1) (38). Given that these MAGs displayed distinct genetic profiles, occupied separate branches in the phylogenetic tree, exhibited low ANI and AAI values, and were coupled with RED values (<1), it was highly plausible that they represented novel species within their respective genera. This discovery of novel species-level MAGs provided valuable insights into the taxonomic diversity and functional complexity of the *E. superba* gut microbiota.

### MAG abundance profile in metagenomic data

To further understand the read relative abundance of 12 MAGs in the *E. superba* gut direct metagenomic data, the reads mapped to those MAGs were collected (Fig. 3C). Among the MAGs, *Yoonia euphausia 2* exhibited the highest read relative abundance at 18.6%, following by *Psychromonas euphausia* (17.6%), *Pseudoalteromonas euphausia* (12.2%). The read relative abundance of *Psychrobacter euphausia 1*, *Psychrobacter euphausia 2*, and *Psychrobacter euphausia 3* were similar, with values of 9.2%, 9.2%, and 9.1%. *Mariniblastus euphausia* displayed a moderate read relative abundance of 8.9%. *Halioglobus euphausia 2* (6.5%) was more abundant than *Halioglobus euphausia 1* (4.1%). *Yoonia euphausia 1* (1.9%) and *Arthrobacter euphausia 1* (1.7%) had the lowest abundances alongside with *Arthrobacter euphausia 2* (1.1%). These findings revealed variation in the read relative abundance of gut microbes in *E. superba*, which might reflect their varying ecological roles.

The high read relative abundance of certain MAGs underscored their ecological significance within the gut microbiome and suggested that they might play essential roles in the metabolic and physiological processes to sustain host homeostasis (39, 40). Moreover, abundant microbes often played critical roles in nutrient synthesis, such as the production of vitamins and amino acids, which were vital for the host growth, health, and immune functions (41–43). These abundant microbes also established stable, long-term relationships with the host, co-evolving to fulfill specific ecological roles (44). Thereby, it could be hypothesized that the high read relative abundance of MAGs might be correlated with their critical contributions to nutrient acquisition and microbial community stability, which, in turn, supported the survival of *E. superba* in extreme environment.

### Metabolic overview

**Fig 4:**
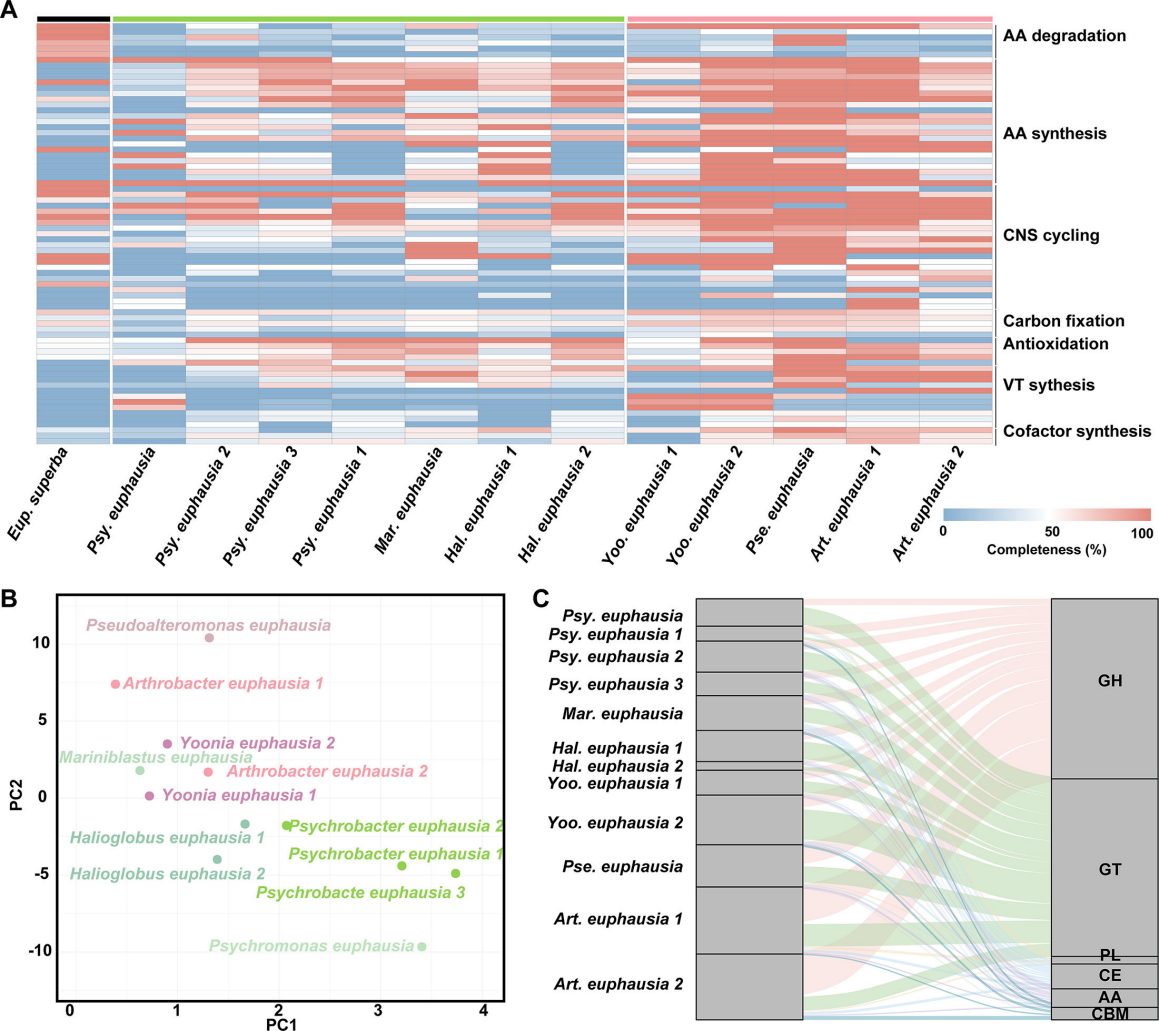
Completeness of material synthesis modules, principal component analysis (PCA), and carbohydrate-hydrolyzing enzymes composition among 12 MAGs. (**A**) Heat map of expressed functional genes (KO) involved in nutrients synthesis modules listed in the Table S4. (**B**) The Euclidean distance across 12 MAGs based on the completeness of metabolic modules. (**C**) The Sankey diagram of carbohydrate-hydrolyzing enzymes in each MAG. The carbohydrate-hydrolyzing enzymes genes included GH, GT, PL, CE, AA, and CBM.

Based on the annotation results, the completeness of metabolic modules in each MAG was assessed (Fig. 4A). The PCA revealed that microbes in same genus had close Euclidean distances, which might reflect evolutionary conservation of metabolic functions, albeit with varying ecological importance possibly driven by differences in read relative abundance (Fig. 4B) (45). The hierarchical clustering indicated that MAGs from the same genus exhibited tightly clustered, also suggesting similar metabolic characteristics and functional profiles (Fig. S5). Additionally, the MAGs could be grouped into two distinct hierarchical clusters based on their completeness of metabolic modules, which they all were far from *E. superba* (Fig. S5). The microbial cluster 1 contained *Psychromonas euphausia*, *Psychrobacter euphausia 1*, *Psychrobacter euphausia 2*, *Psychrobacter euphausia 3*, *Mariniblastus euphausia*, *Halioglobus euphausia 1*, and *Halioglobus euphausia 2*, while the microbial cluster 2 comprised *Yoonia euphausia 1*, *Yoonia euphausia 2*, *Pseudoalteromonas euphausia*, *Arthrobacter euphausia 1*, and *Arthrobacter euphausia 2*. Such division highlighted the functional diversity in the gut microbiota, which might contribute to the homeostasis of the *E. superba* (46).

**Fig 5:**
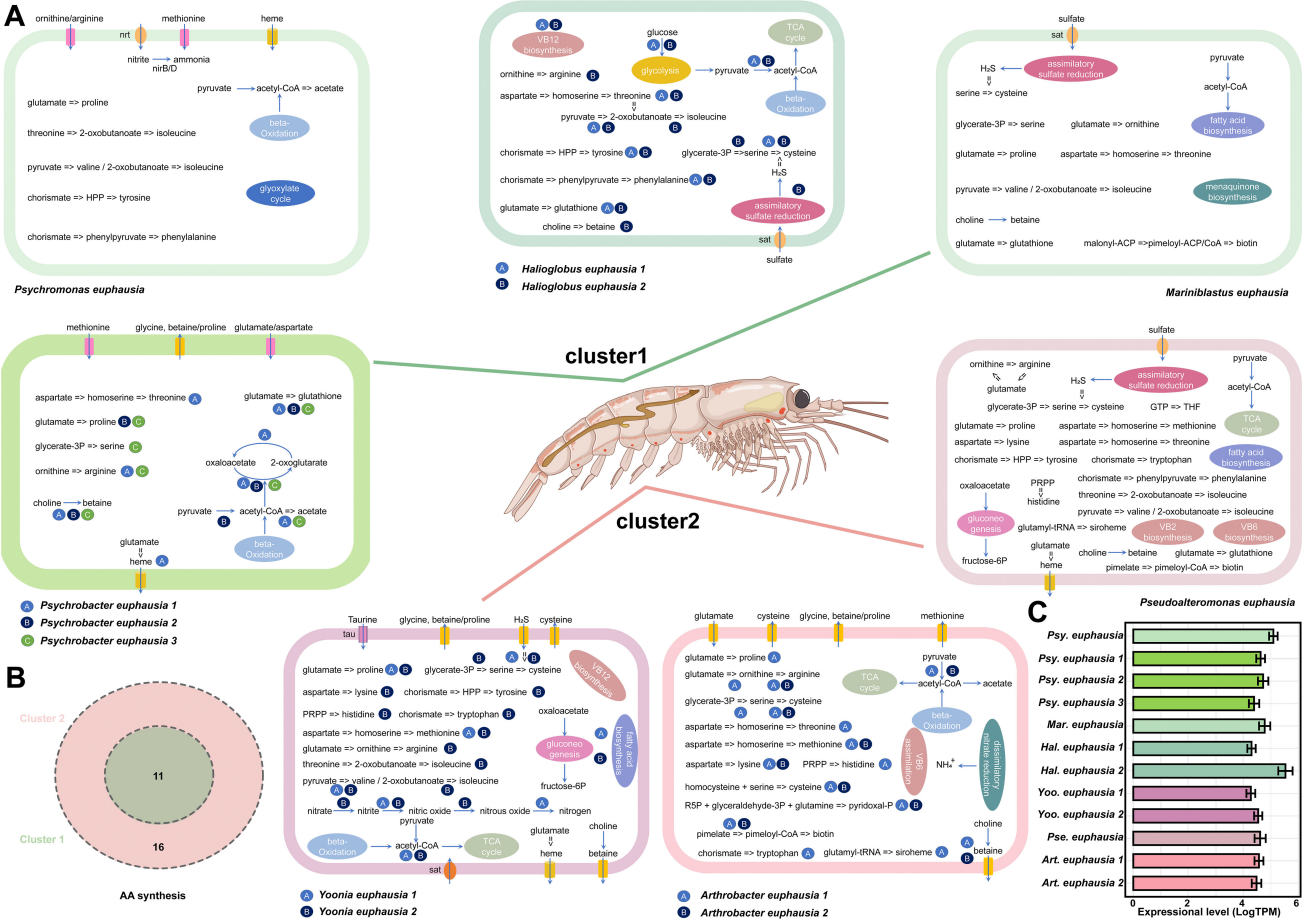
Metabolic diagram of the gut microbiota in *E. superba*. (**A**) The proposed schematic views of the metabolic traits of 12 MAGs. (**B**) The Venn diagram of amino acids synthesis between cluster 1 and cluster 2. (**C**) The expressional level of 12 MAGs.

The *E. superba* harbored metabolic pathways that enabled the degradation of certain amino acids, such as histidine, methionine, tyrosine, and leucine, but lacked their synthetic modules. However, microbes in cluster 1 were capable of synthesizing 11 amino acids, 8 of which could not be synthesized by the host. In contrast, microbes in cluster 2 had the ability to synthesize 16 amino acids, of which 13 amino acids were not synthesized by *E. superba* (Fig. 4A, 5A and B; Table S4). Although microbes in cluster 1 exhibited higher read relative abundance compared to those in cluster 2 (Wilcox-test, *P* < 0.05), the latter displayed a broader range of complete metabolic modules, highlighting their potentially significant role in supplementing essential amino acids required by the host. Moreover, microbes in cluster 2 possessed a higher number of carbohydrate-hydrolyzing enzymes than those in cluster 1, among which GHs and CBMs displayed statistically significant differences between the two clusters (Wilcox-test, *P* < 0.05) (Fig. 4C). However, the presence of carbohydrate-hydrolyzing enzymes exhibited no significant expression differences in 12 MAGs, suggesting they might contribute collectively to the organic digestion processes in *E. superba* (Fig. S6) (11).

The extreme Antarctic environment, characterized by low temperatures and high oxygen levels, posed significant challenges for organisms by elevating production of reactive oxygen species (ROS) (47). To counteract oxidative damage induced by low-temperature stress, antioxidants played critical roles in enhancing the resilience and survival of Antarctic organisms (48–50). Microbes in both clusters, with the exception of *Psychromonas euphausia*, *Arthrobacter euphausia 1*, and *Arthrobacter euphausia 2*, exhibited the capability to synthesize glutathione (Fig. 4A and 5A). Similarly, microbes across all clusters had nearly complete heme and ubiquinone synthesis modules. Those compounds might be served as nonenzymatic antioxidants to support *E. superba* in mitigating the challenges of the Antarctic environment (Fig. 4A and 5A) (51). Notably, microbes, except for *Mariniblastus euphausia 1*, *Psychromonas euphausia*, and *Halioglobus euphausia 1*, demonstrated the ability to synthesize betaine as important osmoregulator to protect *E. superba* from extreme environment (Fig. 4A and 5A) (52). Moreover, microbes in cluster 2 also possessed the ability to synthesize multiple vitamins (Fig. 4A and 5A). For example, *Pseudoalteromonas euphausia* had nearly complete modules for the synthesis of riboflavin, biotin, and pyridoxal-P. *Yoonia euphausia 1* and *Yoonia euphausia 2* could synthesize cobalamin, while *Arthrobacter euphausia 1* and *Arthrobacter euphausia 2* were capable of synthesizing pyridoxal-P. Furthermore, genes involved in secretion systems, along with multifunctional transporters, were found to be abundant in 12 MAGs (Table S5), which further supported the hypothesis that microbes could release metabolites for utilization by *E. superba*. Over the course of long-term evolution, mutual dependencies between *E. superba* and gut microbiota might have been established, where the microbes supply essential nutrients to the host, while the host provided stable habitat and resources for microbial persistence.

Further metatranscriptomic analysis provided evidence of transcriptional activity in these 12 dominant bacteria, affirming their functional presence and metabolic contributions in the *E. superba* gut microbiota (Fig. 5C). Interestingly, microbes in cluster 1 exhibited higher transcriptomic activity compared to those in cluster 2 (Wilcox-test, *P* < 0.05), contrasting with their read relative abundance in the direct metagenomic data (Wilcox-test, *P* < 0.05). The divergence further underscored the complementary and functionally partitioned roles of microbes in the two clusters. However, among the top 200 highly expressed genes, 160 genes were classified as unknown proteins hinting that their potential functional roles within the host-microbe ecosystem remain to be further explored (Fig. S7).

### Conclusion

The study provided comprehensive characterization of the *E. superba* gut microbiome, identifying diverse and unique microbial community that might play vital roles in the host metabolism. The combination of direct metagenomic and culture-enriched metagenomic, allowed for the recovery of a broad range of microbial species, some of which were likely not found in previous study. The functional contributions of these microbes included the synthesis of essential amino acids, vitamins, antioxidants, and osmoregulatory compound, all of which were crucial for the host survival in the harsh Antarctic environment. Those findings underscored the intricate co-evolutionary relationship between *E. superba* and their gut microbiota, revealing potential mechanisms by which the microbes aided in nutrient acquisition and environmental adaptation.

## Data Availability

The 16S rRNA gene amplicon, direct metagenomic, culture-enriched metagenomic, and metatranscriptomic data are available in NCBI under BioProject accession number PRJNA1210771.

## SUPPLEMENTAL MATERIAL

Supplemental Material msystems.00377-25-s0001.doc Figures S1–S7.

Supplemental Tables msystems.00377-25-s0002.xls Tables S1–S5.

ASM does not own the copyrights to Supplemental Material that may be linked to, or accessed through, an article. The authors have granted ASM a non-exclusive, world-wide license to publish the Supplemental Material files. Please contact the corresponding author directly for reuse.

## References

[1] Bar-On YM, Phillips R, Milo R. 2018. The biomass distribution on Earth. Proc Natl Acad Sci USA 115:6506–6511. doi:10.1073/pnas.1711842115 29784790 PMC6016768

[2] Hill SL, Murphy EJ, Reid K, Trathan PN, Constable AJ. 2006. Modelling Southern Ocean ecosystems: krill, the food-web, and the impacts of harvesting. Biol Rev Camb Philos Soc 81:581–608. doi:10.1017/S1464793106007123 16987430

[3] Cavan EL, Belcher A, Atkinson A, Hill SL, Kawaguchi S, McCormack S, Meyer B, Nicol S, Ratnarajah L, Schmidt K, Steinberg DK, Tarling GA, Boyd PW. 2019. The importance of Antarctic krill in biogeochemical cycles. Nat Commun 10:4742. doi:10.1038/s41467-019-12668-7 31628346 PMC6800442

[4] Kawaguchi S, Atkinson A, Bahlburg D, Bernard KS, Cavan EL, Cox MJ, Hill SL, Meyer B, Veytia D. 2023. Climate change impacts on Antarctic krill behaviour and population dynamics. Nat Rev Earth Environ 5:43–58. doi:10.1038/s43017-023-00504-y

[5] Manno C, Fielding S, Stowasser G, Murphy EJ, Thorpe SE, Tarling GA. 2020. Continuous moulting by Antarctic krill drives major pulses of carbon export in the north Scotia Sea, Southern Ocean. Nat Commun 11:6051. doi:10.1038/s41467-020-19956-7 33247126 PMC7699634

[6] Belcher A, Henson SA, Manno C, Hill SL, Atkinson A, Thorpe SE, Fretwell P, Ireland L, Tarling GA. 2019. Krill faecal pellets drive hidden pulses of particulate organic carbon in the marginal ice zone. Nat Commun 10:889. doi:10.1038/s41467-019-08847-1 30792498 PMC6385259

[7] Shao C, Sun S, Liu K, Wang J, Li S, Liu Q, Deagle BE, Seim I, Biscontin A, Wang Q, et al.. 2023. The enormous repetitive Antarctic krill genome reveals environmental adaptations and population insights. Cell 186:1279–1294. doi:10.1016/j.cell.2023.02.005 36868220

[8] Ryabov AB, de Roos AM, Meyer B, Kawaguchi S, Blasius B. 2017. Competition-induced starvation drives large-scale population cycles in Antarctic krill. Nat Ecol Evol 1:0177. doi:10.1038/s41559-017-0177 28685164 PMC5495168

[9] Clarke LJ, Suter L, King R, Bissett A, Deagle BE. 2018. Antarctic krill are reservoirs for distinct southern ocean microbial communities. Front Microbiol 9:3226. doi:10.3389/fmicb.2018.03226 30697197 PMC6340936

[10] Clarke LJ, Suter L, King R, Bissett A, Bestley S, Deagle BE. 2021. Bacterial epibiont communities of panmictic Antarctic krill are spatially structured. Mol Ecol 30:1042–1052. doi:10.1111/mec.15771 33300251

[11] Möller L, Vainshtein Y, Meyer B, Neidhardt J, Eren AM, Sohn K, Rabus R. 2024. Rich microbial and depolymerising diversity in Antarctic krill gut. Microbiol Spectr 12:e0403523. doi:10.1128/spectrum.04035-23 38466097 PMC10986584

[12] Cui X, Zhu G, Liu H, Jiang G, Wang Y, Zhu W. 2016. Diversity and function of the Antarctic krill microorganisms from Euphausia superba. Sci Rep 6:36496. doi:10.1038/srep36496 27812046 PMC5095602

[13] Feng Z, Wang Y, Ma L, Huang S, Wang L, He J, Guo C. 2023. Genomic characteristics and functional analysis of Brucella sp. strain WY7 isolated from Antarctic krill. Microorganisms 11:2281. doi:10.3390/microorganisms11092281 37764125 PMC10536100

[14] Wang Y, Xie J, Feng Z, Ma L, Wu W, Guo C, He J. 2024. Genomic insights into the cold adaptation and secondary metabolite potential of Pseudoalteromonas sp. WY3 from Antarctic krill. Front Microbiol 15:1459716. doi:10.3389/fmicb.2024.1459716 39564484 PMC11573776

[15] Bolyen E, Rideout JR, Dillon MR, Bokulich NA, Abnet CC, Al-Ghalith GA, Alexander H, Alm EJ, Arumugam M, Asnicar F, et al.. 2019. Reproducible, interactive, scalable and extensible microbiome data science using QIIME 2. Nat Biotechnol 37:852–857. doi:10.1038/s41587-019-0209-9 31341288 PMC7015180

[16] Quast C, Pruesse E, Yilmaz P, Gerken J, Schweer T, Yarza P, Peplies J, Glöckner FO. 2013. The SILVA ribosomal RNA gene database project: improved data processing and web-based tools. Nucleic Acids Res 41:D590–D596. doi:10.1093/nar/gks1219 23193283 PMC3531112

[17] Bastian M, Heymann S, Jacomy M. 2009. Gephi: an open source software for exploring and manipulating networks. ICWSM 3:361–362. doi:10.1609/icwsm.v3i1.13937

[18] Li H. 2013. Aligning sequence reads, clone sequences and assembly contigs with BWA-MEM. arXiv. doi:10.48550/arXiv.1303.3997

[19] Chen S. 2023. Ultrafast one-pass FASTQ data preprocessing, quality control, and deduplication using fastp. Imeta 2:e107. doi:10.1002/imt2.107 38868435 PMC10989850

[20] Li D, Liu C-M, Luo R, Sadakane K, Lam T-W. 2015. MEGAHIT: an ultra-fast single-node solution for large and complex metagenomics assembly via succinct de Bruijn graph. Bioinformatics 31:1674–1676. doi:10.1093/bioinformatics/btv033 25609793

[21] Uritskiy GV, DiRuggiero J, Taylor J. 2018. MetaWRAP-a flexible pipeline for genome-resolved metagenomic data analysis. Microbiome 6:158. doi:10.1186/s40168-018-0541-1 30219103 PMC6138922

[22] Parks DH, Imelfort M, Skennerton CT, Hugenholtz P, Tyson GW. 2015. CheckM: assessing the quality of microbial genomes recovered from isolates, single cells, and metagenomes. Genome Res 25:1043–1055. doi:10.1101/gr.186072.114 25977477 PMC4484387

[23] Chaumeil P-A, Mussig AJ, Hugenholtz P, Parks DH. 2019. GTDB-Tk: a toolkit to classify genomes with the Genome Taxonomy Database. Bioinformatics 36:1925–1927. doi:10.1093/bioinformatics/btz848 31730192 PMC7703759

[24] Olm MR, Brown CT, Brooks B, Banfield JF. 2017. dRep: a tool for fast and accurate genomic comparisons that enables improved genome recovery from metagenomes through de-replication. ISME J 11:2864–2868. doi:10.1038/ismej.2017.126 28742071 PMC5702732

[25] Seemann T. 2014. Prokka: rapid prokaryotic genome annotation. Bioinformatics 30:2068–2069. doi:10.1093/bioinformatics/btu153 24642063

[26] Drula E, Garron M-L, Dogan S, Lombard V, Henrissat B, Terrapon N. 2022. The carbohydrate-active enzyme database: functions and literature. Nucleic Acids Res 50:D571–D577. doi:10.1093/nar/gkab1045 34850161 PMC8728194

[27] Capella-Gutiérrez S, Silla-Martínez JM, Gabaldón T. 2009. trimAl: a tool for automated alignment trimming in large-scale phylogenetic analyses. Bioinformatics 25:1972–1973. doi:10.1093/bioinformatics/btp348 19505945 PMC2712344

[28] Nguyen L-T, Schmidt HA, von Haeseler A, Minh BQ. 2015. IQ-TREE: a fast and effective stochastic algorithm for estimating maximum-likelihood phylogenies. Mol Biol Evol 32:268–274. doi:10.1093/molbev/msu300 25371430 PMC4271533

[29] Jain C, Rodriguez-R LM, Phillippy AM, Konstantinidis KT, Aluru S. 2018. High throughput ANI analysis of 90K prokaryotic genomes reveals clear species boundaries. Nat Commun 9:5114. doi:10.1038/s41467-018-07641-9 30504855 PMC6269478

[30] Zhou Z, Tran PQ, Kieft K, Anantharaman K. 2020. Genome diversification in globally distributed novel marine Proteobacteria is linked to environmental adaptation. ISME J 14:2060–2077. doi:10.1038/s41396-020-0669-4 32393808 PMC7367891

[31] Liu C, Chen X, Hu M, Waiho K, Xiao Y, Shang Y, Gao T, Wang Y. 2025. Energy reserves and gut microbiota of marine mussels under combined exposure to pathogens and predation risk. Aquaculture 595:741483. doi:10.1016/j.aquaculture.2024.741483

[32] Manrique-de-la-Cuba MF, Parada-Pozo G, Rodríguez-Marconi S, López-Rodríguez MR, Abades S, Trefault N. 2024. Evidence of habitat specificity in sponge microbiomes from Antarctica. Environ Microbiome 19:100. doi:10.1186/s40793-024-00648-4 39633476 PMC11619120

[33] Robles-Malagamba MJ, Walsh MT, Ahasan MS, Thompson P, Wells RS, Jobin C, Fodor AA, Winglee K, Waltzek TB. 2020. Characterization of the bacterial microbiome among free-ranging bottlenose dolphins (Tursiops truncatus). Heliyon 6:e03944. doi:10.1016/j.heliyon.2020.e03944 32577542 PMC7305398

[34] Shi Y, Wang G, Lau H-H, Yu J. 2022. Metagenomic sequencing for microbial DNA in human samples: emerging technological advances. Int J Mol Sci 23:2181. doi:10.3390/ijms23042181 35216302 PMC8877284

[35] Whelan FJ, Waddell B, Syed SA, Shekarriz S, Rabin HR, Parkins MD, Surette MG. 2020. Culture-enriched metagenomic sequencing enables in-depth profiling of the cystic fibrosis lung microbiota. Nat Microbiol 5:379–390. doi:10.1038/s41564-019-0643-y 31959969

[36] Wang H, Wang M, Fan S, Lu J, Lan Y, Li M, Li J, Liu R, Sun J, Fang J, Qian P-Y, Zhang Y-Z, Zhang W. 2021. Culture enrichment combined with long-read sequencing facilitates genomic understanding of hadal sediment microbes. Front Mar Sci 8:754332. doi:10.3389/fmars.2021.754332

[37] Bowers RM, Kyrpides NC, Stepanauskas R, Harmon-Smith M, Doud D, Reddy TBK, Schulz F, Jarett J, Rivers AR, Eloe-Fadrosh EA, et al.. 2018. Minimum information about a single amplified genome (MISAG) and a metagenome-assembled genome (MIMAG) of bacteria and archaea. Nat Biotechnol 36:660–660. doi:10.1038/nbt0718-660a PMC7608355 29979671

[38] Parks DH, Chuvochina M, Waite DW, Rinke C, Skarshewski A, Chaumeil P-A, Hugenholtz P. 2018. A standardized bacterial taxonomy based on genome phylogeny substantially revises the tree of life. Nat Biotechnol 36:996–1004. doi:10.1038/nbt.4229 30148503

[39] Wei T-S, Gao Z-M, Gong L, Li Q-M, Zhou Y-L, Chen H-G, He L-S, Wang Y. 2023. Genome-centric view of the microbiome in a new deep-sea glass sponge species Bathydorus sp. Front Microbiol 14:1078171. doi:10.3389/fmicb.2023.1078171 36846759 PMC9944714

[40] Liu Y, Zhang Z, Ji M, Hu A, Wang J, Jing H, Liu K, Xiao X, Zhao W. 2022. Comparison of prokaryotes between Mount Everest and the Mariana Trench. Microbiome 10:215. doi:10.1186/s40168-022-01403-y 36476562 PMC9727886

[41] Lan Y, Sun J, Chen C, Sun Y, Zhou Y, Yang Y, Zhang W, Li R, Zhou K, Wong WC, Kwan YH, Cheng A, Bougouffa S, Van Dover CL, Qiu J-W, Qian P-Y. 2021. Hologenome analysis reveals dual symbiosis in the deep-sea hydrothermal vent snail Gigantopelta aegis. Nat Commun 12:1165. doi:10.1038/s41467-021-21450-7 33608555 PMC7895826

[42] Jiang Q, Lin L, Xie F, Jin W, Zhu WY, Wang M, Qiu Q, Li Z, Liu J, Mao S. 2022. Metagenomic insights into the microbe-mediated B and K_2_ vitamin biosynthesis in the gastrointestinal microbiome of ruminants. Microbiome 10:109. doi:10.1186/s40168-022-01298-9 35864536 PMC9306216

[43] Hinzke T, Kleiner M, Breusing C, Felbeck H, Häsler R, Sievert SM, Schlüter R, Rosenstiel P, Reusch TBH, Schweder T, Markert S. 2019. Host-microbe interactions in the chemosynthetic Riftia pachyptila symbiosis. mBio 10:e02243. doi:10.1128/mBio.02243-19 31848270 PMC6918071

[44] de Jonge N, Carlsen B, Christensen MH, Pertoldi C, Nielsen JL. 2022. The gut microbiome of 54 mammalian species. Front Microbiol 13:886252. doi:10.3389/fmicb.2022.886252 35783446 PMC9246093

[45] Louca S, Polz MF, Mazel F, Albright MBN, Huber JA, O’Connor MI, Ackermann M, Hahn AS, Srivastava DS, Crowe SA, Doebeli M, Parfrey LW. 2018. Function and functional redundancy in microbial systems. Nat Ecol Evol 2:936–943. doi:10.1038/s41559-018-0519-1 29662222

[46] The Human Microbiome Project Consortium. 2012. Structure, function and diversity of the healthy human microbiome. Nature 486:207–214. doi:10.1038/nature11234 22699609 PMC3564958

[47] Lu Z, Hoogakker BAA, Hillenbrand C-D, Zhou X, Thomas E, Gutchess KM, Lu W, Jones L, Rickaby REM. 2016. Oxygen depletion recorded in upper waters of the glacial Southern Ocean. Nat Commun 7:11146. doi:10.1038/ncomms11146 27029225 PMC4821880

[48] Song X, Kong F, Liu B-F, Song Q, Ren N-Q, Ren H-Y. 2024. Antioxidants alleviated low-temperature stress in microalgae by modulating reactive oxygen species to improve lipid production and antioxidant defense. Bioresour Technol 413:131451. doi:10.1016/j.biortech.2024.131451 39244108

[49] Zhang L, Zhang Z, Cao J, Wang K, Qin L, Sun Y, Ju W, Qu C, Miao J. 2023. Extreme environmental adaptation mechanisms of Antarctic bryophytes are mainly the activation of antioxidants, secondary metabolites and photosynthetic pathways. BMC Plant Biol 23:399. doi:10.1186/s12870-023-04366-w 37605165 PMC10464054

[50] Bakiu R, Piva E, Pacchini S, Santovito G. 2024. Antioxidant systems in extremophile marine fish species. J Mar Sci Eng 12:1280. doi:10.3390/jmse12081280

[51] He L, He T, Farrar S, Ji L, Liu T, Ma X. 2017. Antioxidants maintain cellular redox homeostasis by elimination of reactive oxygen species. Cell Physiol Biochem 44:532–553. doi:10.1159/000485089 29145191

[52] Ma Y, Wang Q, Gao X, Zhang Y. 2017. Biosynthesis and uptake of glycine betaine as cold-stress response to low temperature in fish pathogen Vibrio anguillarum. J Microbiol 55:44–55. doi:10.1007/s12275-017-6370-2 28035596

