# Supplementary material for: Metabolic potentials of the gut microbes in Antarctic krill (Euphausia superba)
Source: mSystems. 2025 Aug 7;10(9):e00377-25. doi: 10.1128/msystems.00377-25 (PMC12456004; doi:10.1128/msystems.00377-25)
Supplement: Supplemental Material — Figures S1–S7. [file msystems.00377-25-s0001.doc]

**Supplemental information**

**Supplemental Figures**


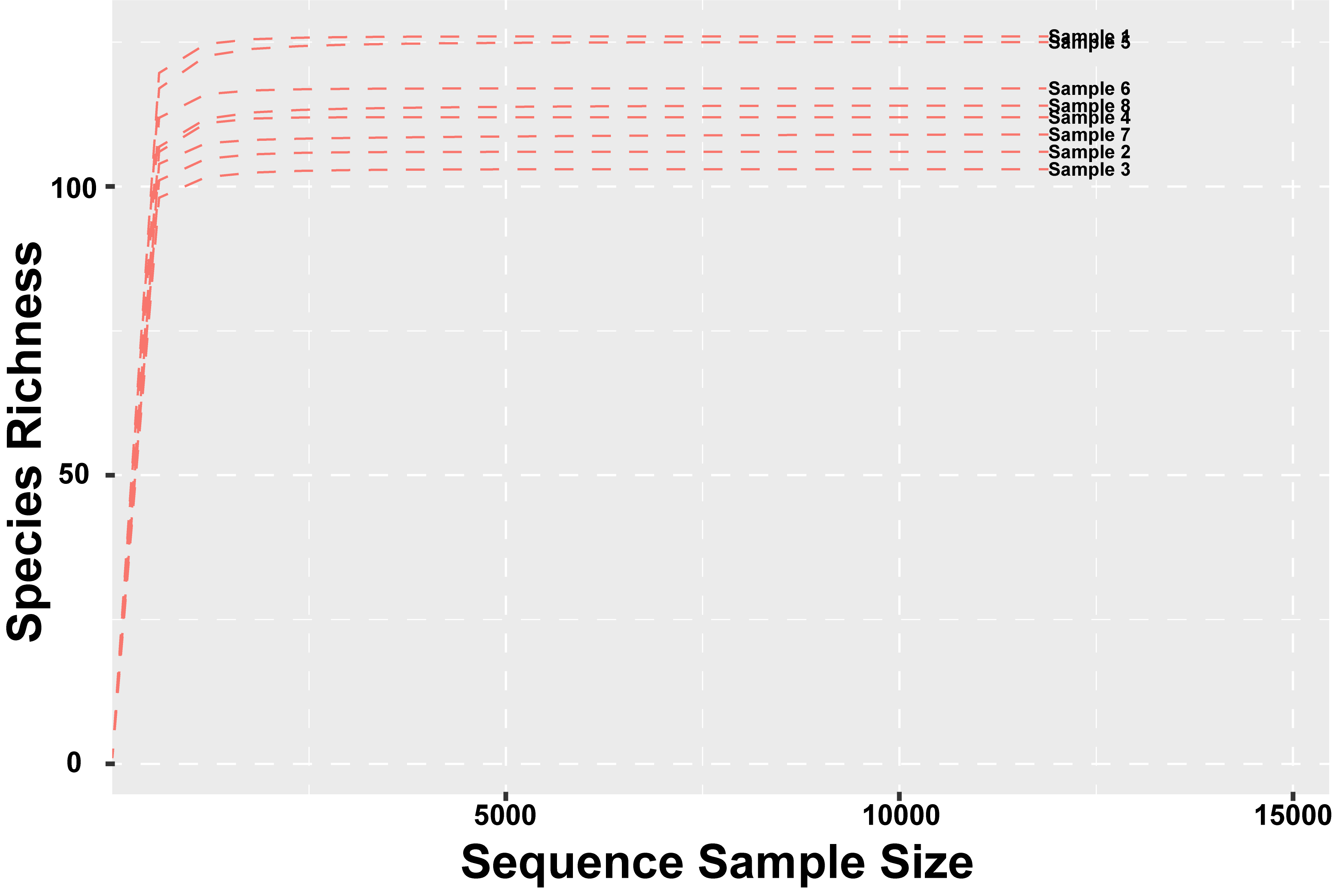


Fig. S1 The rarefaction curves of the species richness observed in the Antarctic krill (*Euphausia superba*) gut microbiota.


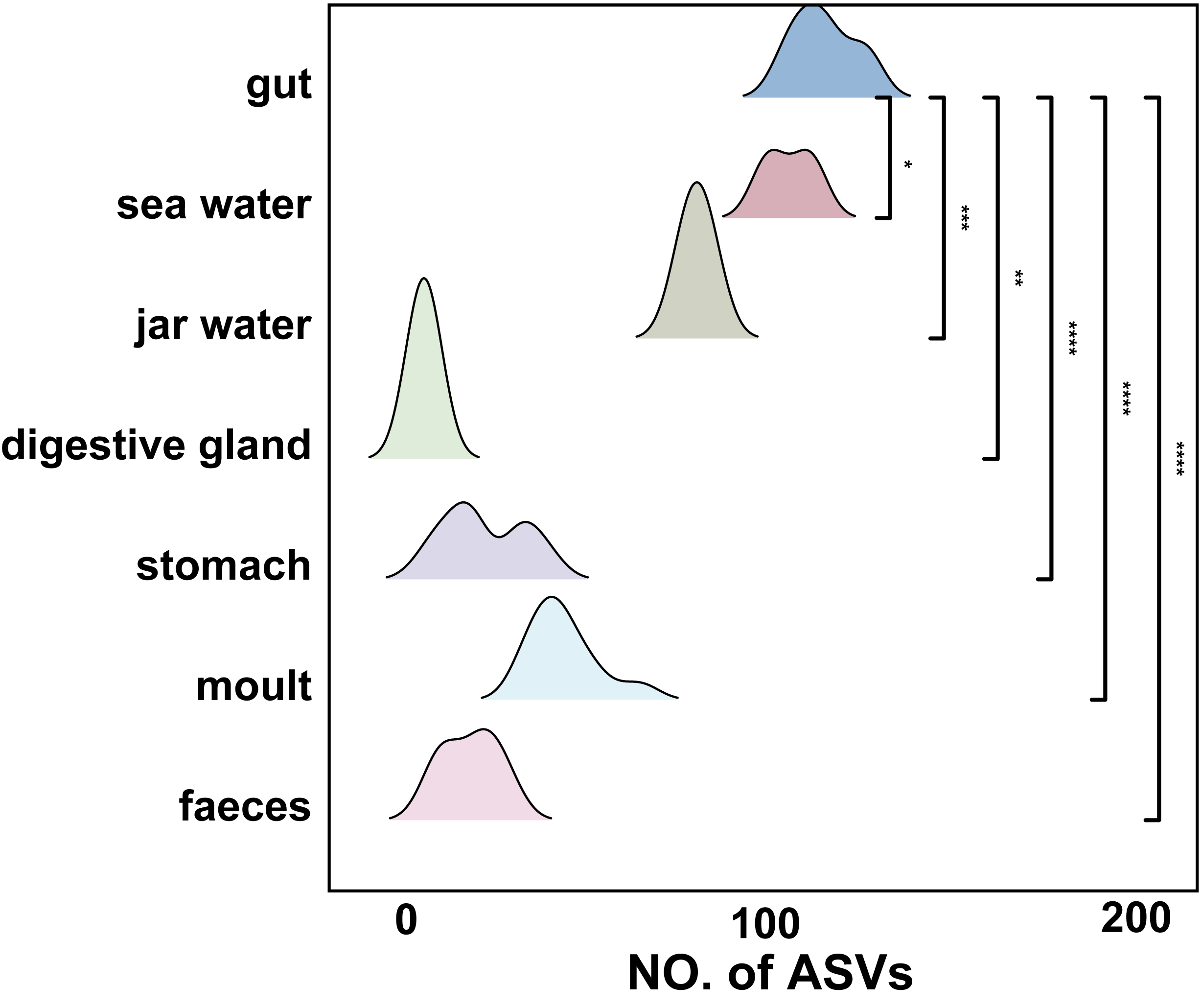


Fig. S2 The number of amplicon sequence variants (ASVs) observed in each sample associated with *E. superba*. * *P* < 0.05, ** *P* < 0.01, *** *P* < 0.001, **** *P* < 0.001.


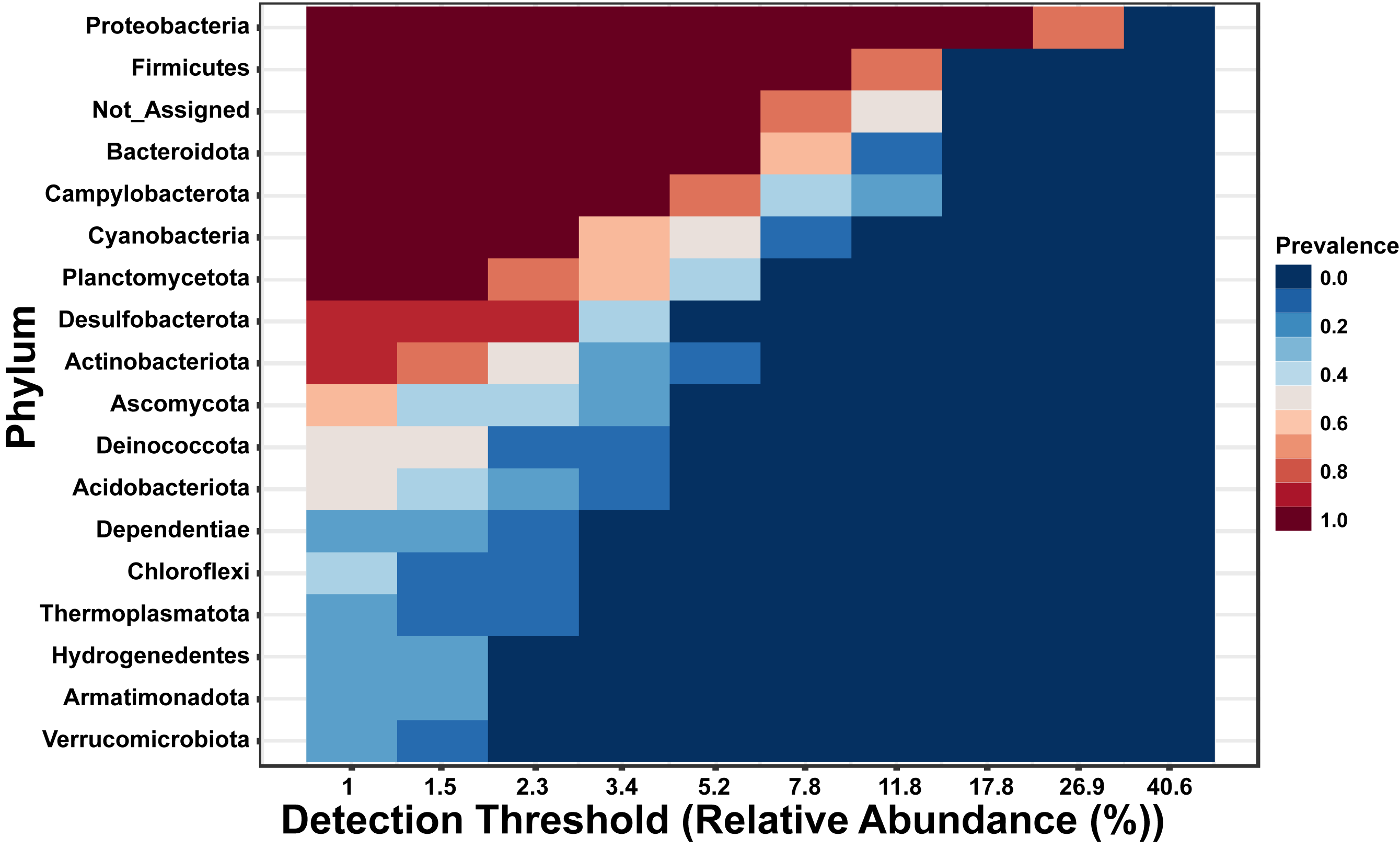


Fig. S3 The core microbes within the *E. superba* gut microbiota.


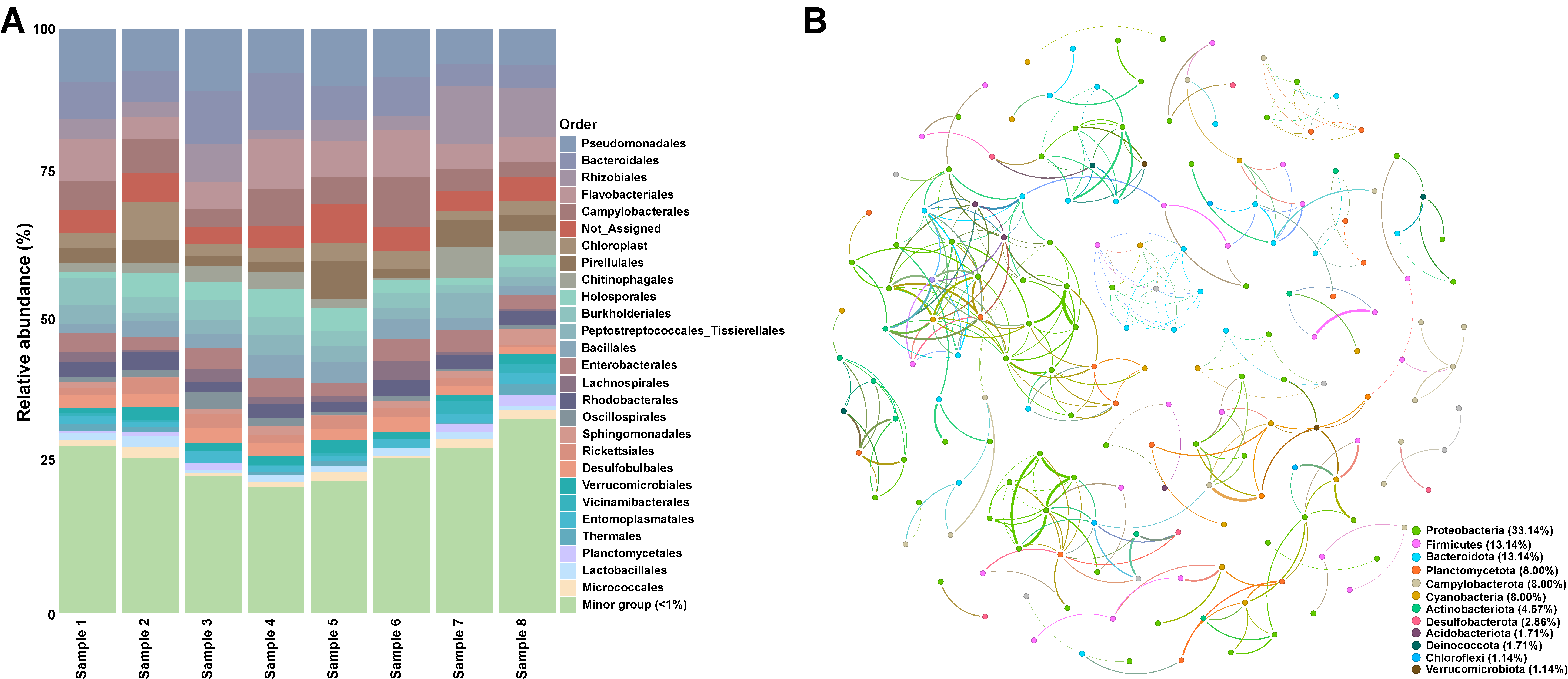


Fig. S4 The gut microbial composition at order and co-occurrence patterns in the *E. superba* gut microbiome.

A) The gut microbial composition at order observed in eight *E. superba*. B) The co-occurrence patterns in the *E. superba* gut microbiota revealed by network analysis at the phylum level. Connections represented significant correlations between species (ρ > 0.8, *P* < 0.05). The highly abundant ASVs were listed in the Table S2.


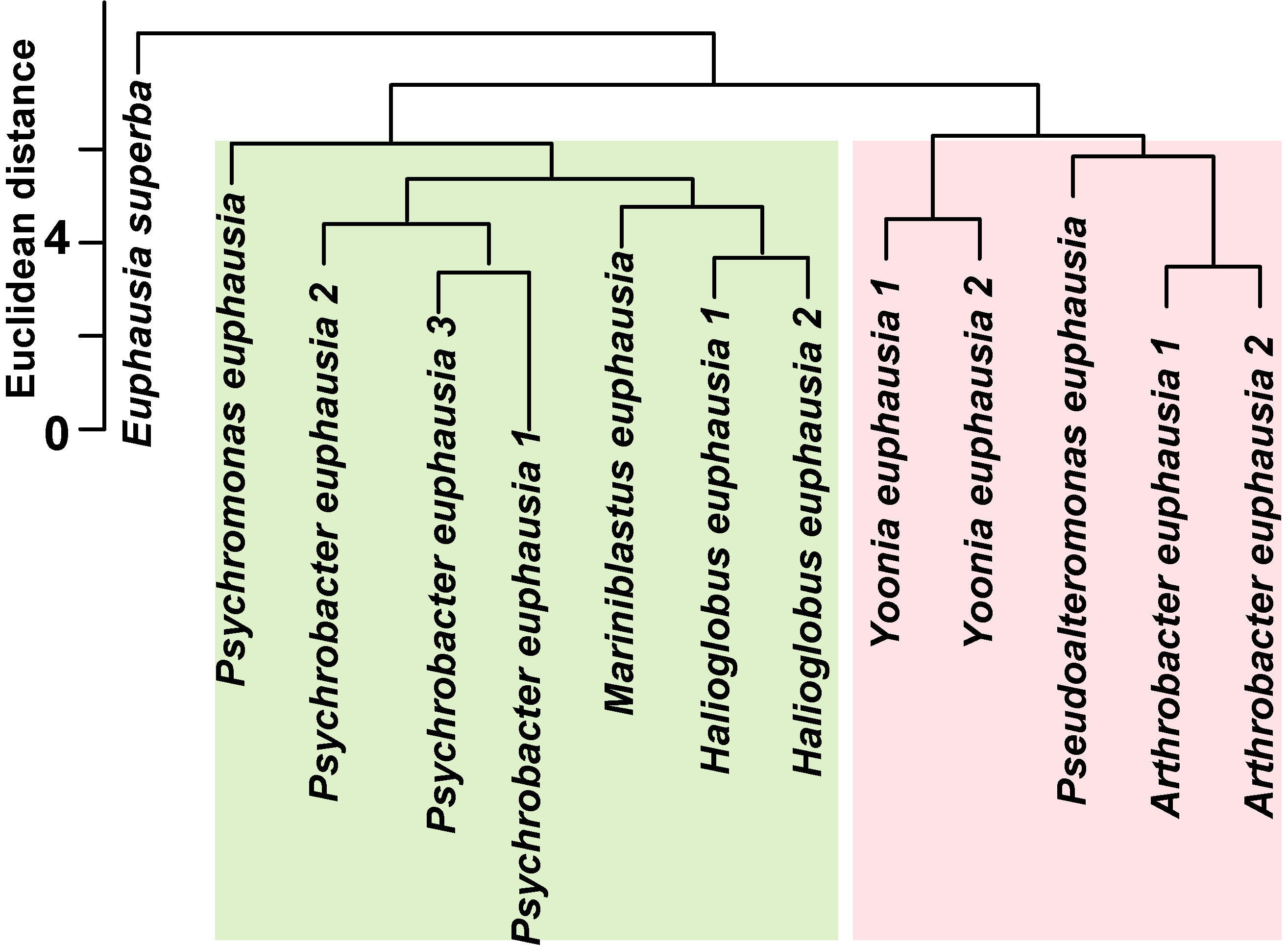


Fig. S5 The Euclidean distances based on the completeness of material synthesis modules among *E. superba* genome and 12 metagenome-assembled genomes (MAGs). The data was listed in Table S4.


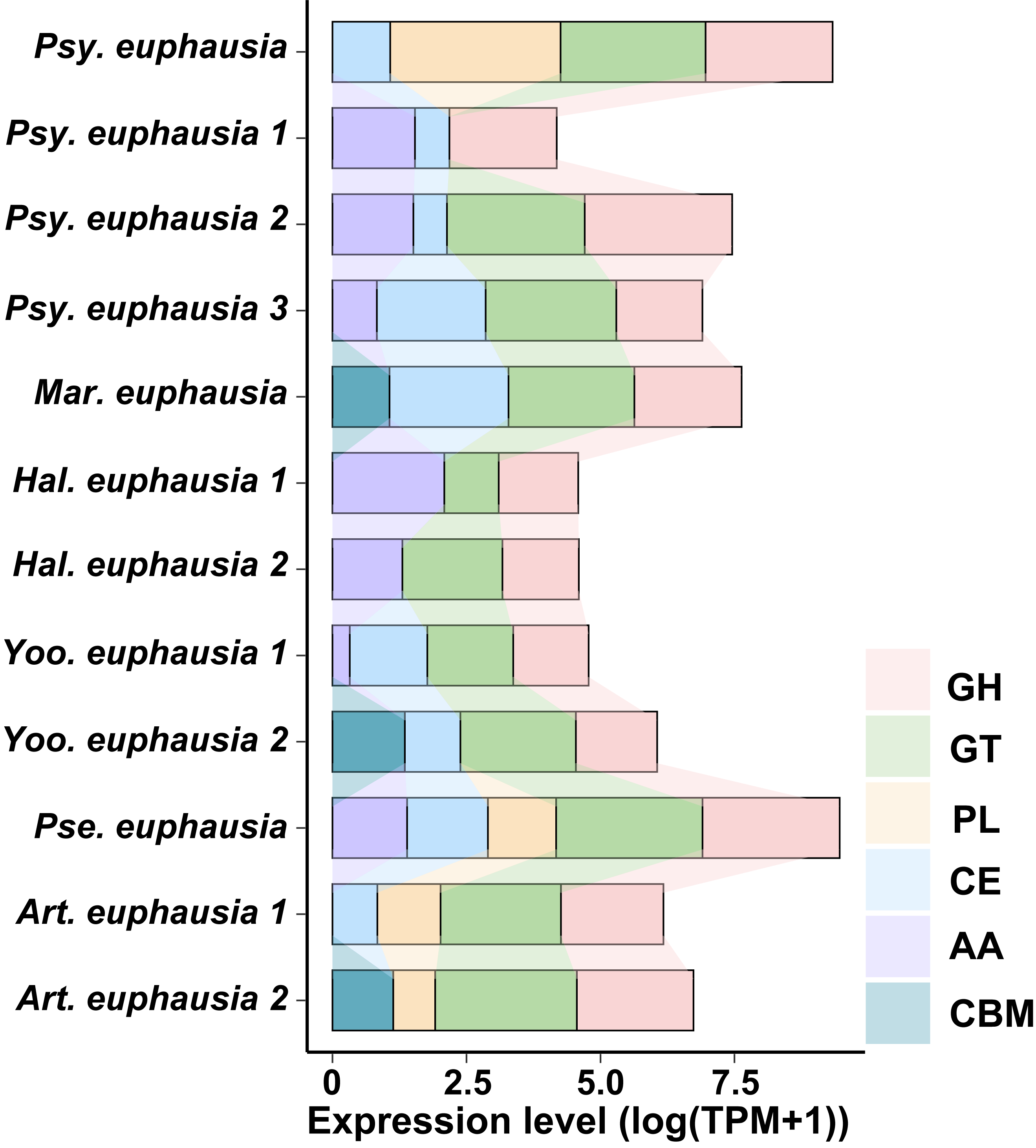


Fig. S6 The expression levels of carbohydrate-hydrolyzing enzymes genes, including GH, GT, PL, CE, AA, and CBM, in the 12 MAGs.


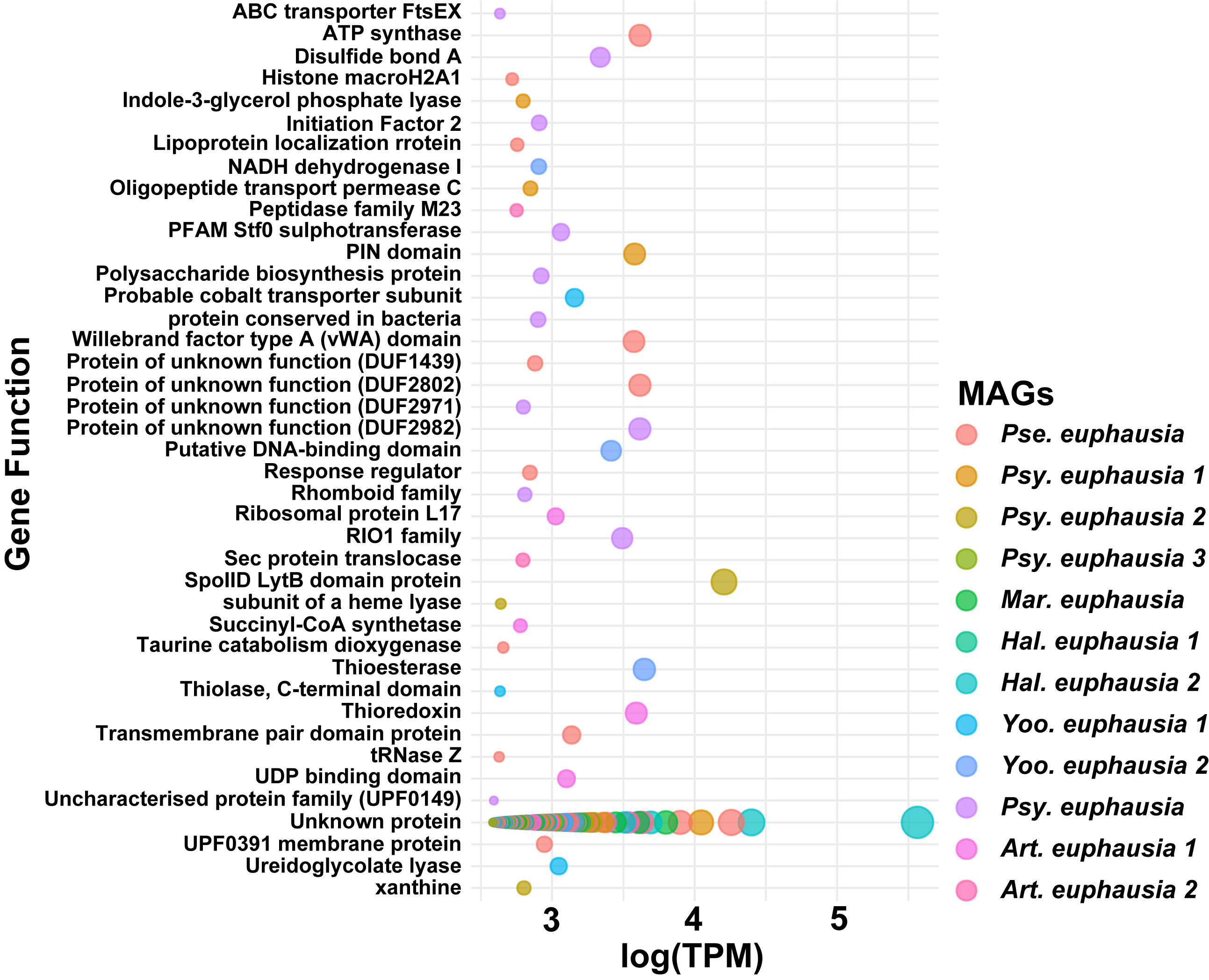


Fig. S7 Top 200 most highly expressed functional genes from 12 MAGs.
